# Awareness and management of elevated blood pressure among human immunodeficiency virus–infected adults receiving antiretroviral therapy in urban Zambia: a call to action

**DOI:** 10.1080/16549716.2017.1359923

**Published:** 2017-10-09

**Authors:** Sophie Bauer, Mwanza Wa Mwanza, Roma Chilengi, Charles B. Holmes, Zude Zyambo, Hansjakob Furrer, Matthias Egger, Gilles Wandeler, Michael J. Vinikoor

**Affiliations:** ^a^ Department of Infectious Diseases, Bern University Hospital, University of Bern, Bern, Switzerland; ^b^ Centre for Infectious Diseases Research in Zambia, Lusaka, Zambia; ^c^ School of Medicine, Johns Hopkins University, Baltimore, USA; ^d^ Institute of Social and Preventive Medicine, University of Bern, Bern, Switzerland; ^e^ Department of Medicine, University of Alabama at Birmingham, Birmingham, USA; ^f^ School of Medicine, University of Zambia, Lusaka, Zambia

**Keywords:** Africa, HIV/AIDS, high blood pressure, antiretroviral therapy, non-communicable diseases

## Abstract

The prevalence of high blood pressure (HBP) and hypertension (HTN), awareness of the diagnoses, and use of anti-hypertensive drugs were examined among human immunodeficiency virus (HIV)-infected individuals on antiretroviral therapy (ART) in Zambia’s capital Lusaka. Within a prospective cohort based at two public sector ART clinics, BP was measured at ART initiation and every 6 months thereafter as a routine clinic procedure. Predictors of HBP (systolic BP ≥140 mmHg or diastolic BP ≥90 mmHg) during one year on ART were analyzed using logistic regression, and the proportion with HTN (2+ episodes of HBP >3 months apart) described. A phone survey was used to understand patient awareness of HBP, use of anti-hypertensive drugs, and history of cardiovascular events (CVE; myocardial infarction or stroke). Among 896 cohort participants, 887 (99.0%) had at least one BP measurement, 98 (10.9%) had HBP, and 57 (6.4%) had HTN. Increasing age (10-year increase in age: adjusted odds ratio [AOR] = 1.50; 95% confidence interval [CI] 1.20–1.93), male sex (AOR = 2.33, 95% CI 1.43–3.80), and overweight/obesity (AOR = 4.07; 95% CI 1.94–8.53) were associated with HBP. Among 66 patients with HBP, 35 (53.0%) reported awareness of the condition, and nine (25.7%) of these reported having had a CVE. Only 14 (21.2%) of those reached reported ever taking an anti-hypertensive drug, and one (1.5%) was currently on treatment. These data suggest that major improvements are needed in the management of HBP among HIV-infected individuals in settings such as Zambia.

## Background

Human immunodeficiency virus (HIV)-infected individuals have an increased risk of cardiovascular disease (CVD) compared to the general population [2]. CVD-related mortality is anticipated to increase as HIV-infected patients initiate antiretroviral therapy (ART) at CD4+ counts >200 and opportunistic infections become uncommon [3–5]. In sub-Saharan Africa, there are numerous challenges in the diagnosis and control of hypertension (HTN), including low awareness among patients and providers [6], overcrowded clinics, and limited opportunities for HTN screening [7]. Care and treatment programs for HIV present a promising platform to address HTN because care is provided longitudinally, and most clinics provide pharmacy, laboratory, and clinical services as well as counseling and health education [8]. In Zambia, where 12.5% of adults are HIV-positive[9] and adult HTN among the general population is estimated at 35% [10], the Ministry of Health (MoH) recommends routine monitoring of blood pressure (BP) as part of HIV care [11]. This study sought to describe the prevalence, awareness, and management of high BP (HBP) in patients attending public-sector HIV clinics in Lusaka, Zambia.

## Methods

A prospective HIV cohort was established in 2013 at two public-sector clinics in Lusaka to investigate viral hepatitis–HIV co-infection [12,13]. From 1 October 2013, to 15 August 2014, HIV-infected and ART-eligible adults (>18 years old) who initiated ART under MoH guidelines were prospectively enrolled [11]. From 16 August 2014 to 30 September 2015, enrollment was restricted to those with HIV–hepatitis B virus co-infection (defined by a positive hepatitis B surface antigen test).

Data collection mirrored that of the clinics, including baseline assessment of demographic characteristics, World Health Organization HIV clinical stage, and CD4+ count. At ART initiation, potential non-communicable disease (NCD) risk factors were assessed including body mass index, current smoking status (yes/no and pack-years among current smokers), alcohol consumption (based on the Alcohol Use Disorders Identification Test-Consumption), and BP. BP was measured once in the upper arm by a nurse or lay health worker using a battery-operated automated monitoring device (Omron brand, Kyoto, Japan) and medium-sized adult cuff after the patient was seated for 2–4 min. The preferred ART regimen at the time was fixed-dose combination of efavirenz, emtricitabine, and tenofovir disoproxil fumarate. At 24- and 48-week follow-up visits, BP was re-measured per clinic practice. Medications for HTN are free of charge in the outpatient departments of health facilities in Zambia. However, clinicians in ART clinic may also prescribe these drugs.

During December 2015 to January 2016, a phone survey was conducted of all cohort participants with HBP, defined as elevated systolic BP ≥140 mmHg or diastolic BP ≥90 mmHg, during the first year on ART. HTN was defined as elevated systolic or diastolic BP measurements on two separate visits at least 3 months apart. Pharmacy records were also extracted for any anti-hypertensive, any diabetic drugs, or any documented CVD events. Using the phone number on file in the clinic, a nurse made up to five attempted calls to patients with HBP over several different days and times. If reached, using a standardized data collection tool, the nurse asked a series of questions in the local language (Nyanja, Bemba, or English) concerning patient awareness and history of HBP (including location of diagnosis). Prior or current receipt of anti-hypertensive drugs, history of other CVD risk factors (diabetes) or cardiovascular events (CVE; myocardial infarction or stroke), and family history of HTN were ascertained by patient report.

Factors associated with having HBP in the cohort were assessed using a stepwise logistic regression model with a backward selection algorithm. The removal probability was set at 0.2 using likelihood ratio test. Among HBP patients who participated in the phone survey, the proportion with awareness of the condition and the proportion who ever took anti-hypertensive drugs were described, as well as the number who self-reported a history of CVE. Stata v13 (StataCorp, College Station, TX) was used for analysis. The University of Zambia Biomedical Research Ethics Committee approved the study (protocol # 010-08-12; initial approval date 9 November 2012).

## Results

Among 896 cohort participants, the median age was 34 (interquartile range [IQR] 29–40 years), 467 (52.1%) were women, and the median CD4+ count at ART initiation was 227 cells/mm^3^ (IQR 118–337 cells/mm^3^). At baseline, 102 (11.6%) patients were overweight or obese, and this was more common among women than men (17.6% vs. 5.0%; *p* < 0.001). Men were more likely to report smoking (21.7% vs. 2.4%; *p* < 0.001) at baseline compared to women. Patient demographics were similar between 2013–2014 and 2014–2015. During the first year on ART, 60 (6.7%) were lost to follow-up.

BP was measured for 883 (98.5%) at baseline, for 751 (97.9%) of the 767 with retention at the 6-month visit, and for 604 (84.8%) of the 712 with retention at the 12-month visit; 770 (85.8%) had at least two measurements. During the first year on ART, 98 (10.9%) patients had documented HBP on at least one occasion, and 57 (6.4%) had HTN. Retention at 1 year was similar between patients with HBP and normal BP at baseline (86.8% vs. 80.9%; *p* = 0.29). Increasing age (for each 10-year increase in age [1]: adjusted odds ratio [AOR] = 1.50; 95% confidence interval [CI] 1.20–1.93), male sex (AOR = 2.33, 95% CI 1.43–3.80), and overweight/obesity (AOR = 4.07; 95% CI 1.94–8.53) were associated with increased odds of HBP. Pre-ART CD4+ count was not associated with HBP (Table 1). Repeat analysis using HTN as the outcome identified similar risk factors (data not shown). A sensitivity analysis performed after exclusion of HIV–HBV patients recruited after August 2015 had similar results.

**Table 1.** Factors associated with high blood pressure among HIV-infected adults taking antiretroviral therapy in Lusaka, Zambia (*N* = 896)

	Crude odds ratio (95% CI)	Adjusted odds ratio (95% CI)
Age, per 10-year increase	1.62 (1.30–2.02)	1.52 (1.20–1.93)
Male sex	2.00 (1.30–3.07)	2.33 (1.43–3.80)
Monthly household income		
<K500.00	Reference	
≥K500.00	1.40 (0.84–2.32)	
Education level completed		
No formal education	Reference	
Grade 1–6	0.98 (0.30–3.15)	
Grade 7–9	1.32 (0.45–3.83)	
Grade 10 and above	1.58 (0.53–4.73)	
WHO clinical stage		
1 or 2	Reference	
3 or 4	0.99 (0.65–1.51)	
Body mass index		
<18.5	Reference	Reference
18.5–25	1.69 (0.97–2.93)	1.55 (0.88–2.74)
>25	3.65 (1.86–7.15)	4.07 (1.94–8.53)
HBV coinfection	1.14 (0.70–1.85)	
CD4+ count, per 50-cell increase	1.00 (0.94–1.07)	
Unhealthy alcohol consumption^a^	1.22 (0.81–1.86)	

^a^Unhealthy alcohol use was defined as ±4 points in men and ±3 points in women on the Alcohol Use Disorders Identification Test-Consumption.

CI, confidence interval; WHO, World Health Organization; HBV, hepatitis B virus; BL, baseline; HIV, human immunodeficiency.

During the phone survey, 92 patients with HBP were called. Of these, 66 (71.7%) were reached, and 26 were not reached because there was no answer (*n* = 15), the line was inactive/invalid (*n* = 5), the patient had died (*n* = 3), or there was no documented phone number (*n* = 3). Thirty-five (53.0%) patients reported being aware of having HBP, and awareness increased with increased number of HBP measurements (35.3% for one and 60% for two to three elevated readings). Most patients (62.9%) became aware of HBP at the ART clinic (62.9%), while others were diagnosed at the clinic’s outpatient department (22.9%) or at a referral hospital (14.3%). The diagnosis of HBP was primarily communicated to patients by nurses (60.0%) and less commonly by a physician (25.7%) or a clinical officer (14.3%).

**Figure 1.**
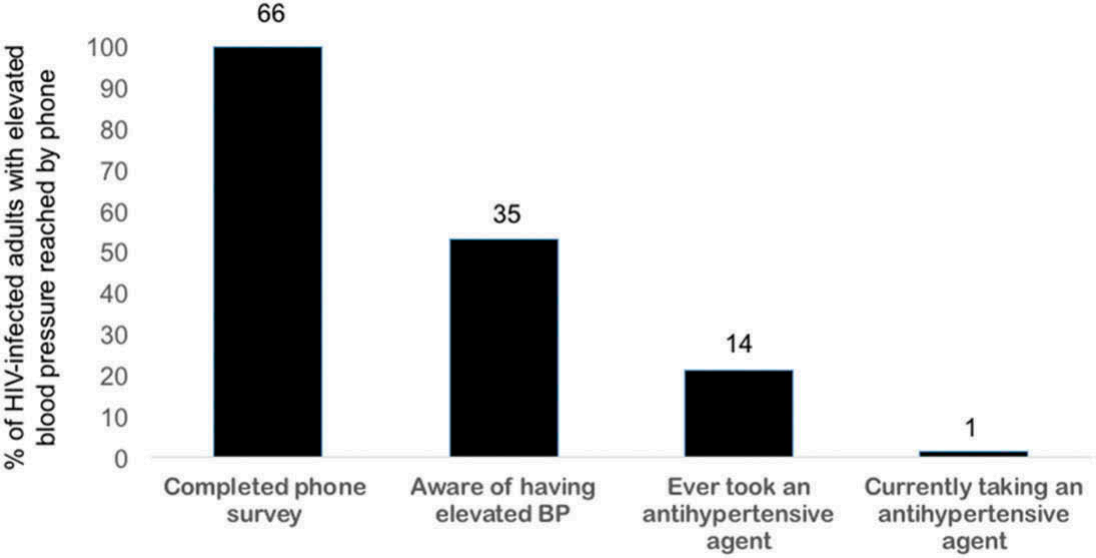
Treatment cascade for human immunodeficiency virus–infected adults with elevated blood pressure and taking antiretroviral therapy in Lusaka, Zambia.

Although many patients were aware of having HBP, only 14 (21.2%) had ever taken an anti-hypertensive drug, and only one person was currently taking a drug for treatment of HTN (Figure 1). Nifedipine (*n* = 4) and furosemide (*n* = 3) were the most commonly taken medicines for HBP, but the majority (*n* = 7) could not recall the names of the drugs they had taken. Review of the HIV clinic records for patients with HBP did not reveal any additional anti-hypertensive prescription or CVE information. Of the 66 patients reached by phone, 24 (36.4%) by either file review or self-report had at least one other known CVD risk factor, and nine (13.6%) self-reported a history of CVE.

## Discussions

To the authors’ knowledge, this is the first study on patient awareness of HBP and its management in an HIV care setting in Zambia. Routinely collected HIV data were augmented using a phone survey, which is an efficient way to assess and distribute public-health information [14]. Approximately 10% had HBP, with 6.4% meeting the criteria for HTN. However, patient awareness of HBP was relatively low (53.0%). Receipt of anti-hypertensive drugs was rare, even among the 13.5% of individuals with reported CVEs, suggesting that major improvements are needed in the management of HTN among HIV-infected individuals in settings such as Zambia.

BP was measured regularly at clinic visits and documented in files, suggesting that screening for HTN in HIV care settings is feasible. The data on HBP build on other studies [15,16] and were significantly lower than a household survey in Lusaka (35%) [10], possibly because of immune suppression (median CD4+ count = 227) and malnourishment (29% had a body mass index <18.5 kg/m^2^) that were common in the cohort. HBP tends to become more common after ART initiation and may cause HTN [4]. Consistent with other African data [17], only half of patients were aware of having HBP, and similar to our study at rural outpatient clinics in Zambia [18], few patients took anti-hypertensive drugs. Challenges to HIV-NCD integration range from low patient and provider awareness, education, and training to structural and logistic barriers [7,19].

This study has several limitations. In the primary analysis, HBP was defined as a single elevated measurement, and BP was measured only once at each time point. This may have inflated the number of patients truly needing treatment, as only 58% of patients with HBP were confirmed to have HTN. Data on currently anti-hypertensive medications were not well documented in ART clinic. Therefore, HTN patients on treatment who did not have HBP during the cohort could have been overlooked. However, this would have been uncommon because so few patients in the phone survey reported taking BP-lowering drugs. Self-reported phone survey data were subject to recall bias and may have been inaccurate. Clinical adjudication of patient-reported CVEs would have strengthened the data, as it is acknowledged that in Zambia, health literacy is low, and reported CVEs may have been miscommunicated. Finally, an urban cohort was studied, and thus the data may not be generalizable to rural areas.

CVD will surpass infectious diseases as the leading cause of death in Africa by 2030 [20], and HTN control should be a major priority for public health on the continent. These data demonstrate that although integration of BP screening and management in HIV care settings was feasible in Zambia, virtually no patient had optimal management of HBP. There is an especially urgent need to test effective interventions to reduce CVD risks among HIV-infected individuals.

## Paper context

In urban Zambia, we assessed the epidemiology of comorbid elevated BP and its management among human immunodeficiency virus-infected individuals receiving antiretroviral therapy. Among 896 analyzed, hypertension was uncommon (<10% prevalence). Among those with at least one high BP measurement, awareness was low, and virtually no patient was taking an anti-hypertensive medication. These data provide a call to action to strengthen diagnosis and management of non-communicable diseases at human immunodeficiency virus care settings in Africa.
